# Headquarters' Failure in India

**Published:** 1919-08-30

**Authors:** 


### The Hospital

The Workers' Newspaper of Administrative Medicine and Institutional Life, Administration, National Insurance and Health.

No. 1734, Vol. LXVI. SATURDAY, AUGUST 30, 1919. PRICE TWOPENCE

### HEADQUARTERS' FAILURE IN INDIA.

The deeper and closer the unpreparedness in Afghanistan is examined, and the more the facts become known, the more complete becomes the exhibition of incompetence at headquarters. This view seems to be held in India too. The latest communication is from the *Times'* own correspondent in Bombay, which was despatched on the 20th and not received by the *Times* until the 26th instant. The correspondent writes of the Government of India, with its customary secrecy, not thinking it worth while to publish their despatches, presumably the White Papers, which should answer the criticism originally made by the Press of India. "Only summaries have so far reached India, where it is held (the *Times of India*) that the despatches do not remove any doubts and do not lead readers to the conclusion that Army Headquarters is doing all it can to bring the true facts to light." The *Civil and Military Gazette* quotes in regard to the lack of provision for cholera treatment "the case of an officer in the Kohat area suffering with cholera dumped down in the middle of a field, and the announcement made that the site would constitute a cholera hospital. The following day a tent was placed over the officer and treatment seriously begun." The *Times* correspondent reports the *Gazette's* comments: "Explanations will not meet the case unless accompanied by drastic action preventing the Army being exposed to such unnecessary sufferings as were endured during the Frontier operations." We have here confirmation of and justification for the good grounds for fear which The Hospital brought to the notice of the Government at home and the people throughout the Empire.

On Saturday last the *Evening Standard*, and on Sunday the *Observer*, printed a Reuter's message from Simla, dated 19th instant, giving the figures and other particulars relating to the cholera outbreak and the number of sick. In this cable we are glad to note that Lieut.-General Sir Charles H. Burtchaell, Director-General of Medical Services, has enabled Reuter's correspondent to give credit to the R.A.M.C.'s arduous but successful work. Here is the testimony: "*In view of these impediments*, the manner in which cholera was suppressed has been a triumph of medical skill and devotion to duty." This just testimony, just because the impediments were some of them appalling, and most or all of them should have been anticipated and provided for, is welcome and has been splendidly earned, as we have demonstrated already in these columns. The grave stupidity and danger of the Indian method of suppression, and the concoction of official *communiqués* for home and Indian consumption, are illustrated in the telegram referred to. The second sentence of it is: "The reports, (of statistics of the sickness of troops on the Frontier), which have reached England are evidently greatly exaggerated." We are becoming so used to Headquarters and Indian methods that we are not surprised to find on examination of the figures, and the accounts previously published that Reuter's correspondent has misunderstood or been posted with figures which support the opposite view to that expressed in his telegram. The figures published in the *Times*, and in The Hospital of August 16, as well as in the Press in England, demonstrate this. Thus in the cholera epidemic in June there were 1,663 cases, most of them amongst the followers, and 566 deaths, of which six were British officers and fifteen Europeans of other ranks. Reuter's telegram on August 19 states: "An unusually severe cholera epidemic broke out at no fewer than thirty stations. It was at its worst in June. There have been seventy-six cases, with thirty deaths, among the British troops, and 2,045 cases and 617 deaths among the Indians. The actual deaths from heat-stroke were twenty-six British and 140 Indians." Here is proof of the conservative character of the figures published by the Press in England up to August 16. There was, it will be seen, no exaggeration, but quite the contrary.

Again, in regard to sickness. On August 16 we published from the *Morning Post* and other papers a belated message made public in India on July 15, which gave the total number of evacuations to hospitals as being British 2,289 and Indian 5,541. These figures included both the Khyber and Kohat lines. The Simla message of the 19th instant—*i.e.*, Reuter's statement to August 9—gives the total admissions to hospital as 10,882 British, and 45,774 Indian troops and followers, with the deaths from disease as 12 officers and 77 other ranks British, and 9 officers and 1,260 other ranks and followers Indian troops. Reuter's statement, according to the correspondent of the *Even-*

*ing Standard* of the 23rd inst., says: "The reports which have reached England are evidently greatly exaggerated." A comparison of the figures in the British Press with the figures given by Reuter's correspondent once more demonstrates that, if there have been reports which have reached England which are "greatly exaggerated" they are not those which have appeared in the columns of the leading Press organs in England.

We reprint on page 539 of The Hospital today an exact copy of a second letter, published in *The Times* of August 26. This contains a description of the Indian Government's mishandling of a grave position affecting a great many of the British medical officers with temporary commissions, who are being badly hit by the treatment that the Indian Government is meting out to them. Every medical reader throughout the Empire will have his sympathies stirred and his wrath awakened at the position of these unfortunate British medical officers. How many members of the profession are there who would like to have an opportunity, for instance, to help and succour the wife of their confrère, who is in financial trouble at home in her husband's absence and has undergone a severe operation, whilst he, poor man, has all his savings spent, his child dead, and his wife ill. All this on the top of a refusal by the Government of India to release and return this poor man home with all possible despatch. If this should reach the eye of any member of the profession, or other reader, who knows the lady in question, and who will communicate with the Editor of The Hospital, speedy relief and aid will be forthcoming, no doubt, without delay or difficulty.

Let all medical readers bear in mind throughout life the reply sent to their middle-aged confrère by those responsible in India, in the form of "an official regret that only urgent cases can be considered." Has any man or woman amongst us an imagination equal to figuring a much more urgent case than his? Is there a single member of the whole profession who will not rejoice in the splendid service rendered by his R.A.M.C. colleagues on the Afghan Frontier? Will not members unite in a sustained effort to secure action, which will retire the men and change the system which have too long tended to dominate medical affairs in India?

